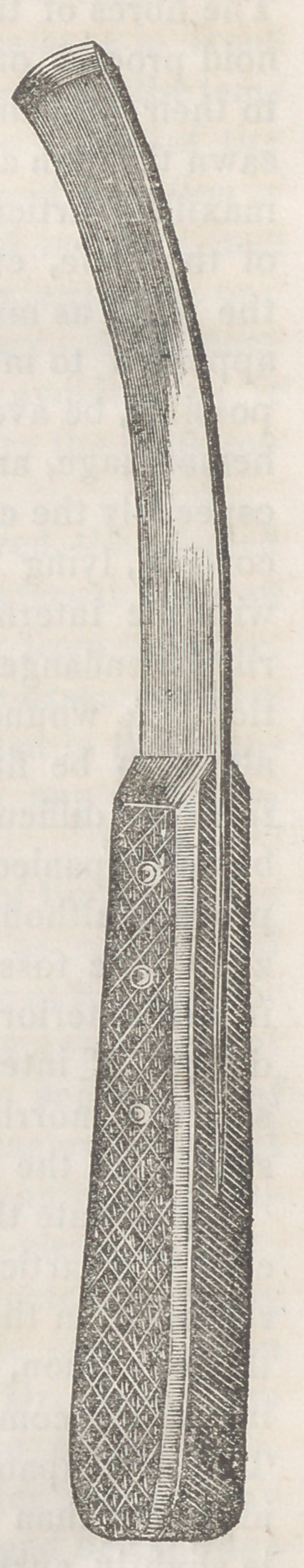# Observations on Excision of the Superior Maxillary Bone; Illustrated by Five Cases

**Published:** 1852-10

**Authors:** S. D. Gross

**Affiliations:** Professor of Surgery in the Medical Department of the University of Louisville


### WESTERN JOURNAL

OF

### MEDICINE AND SURGERY.

OCTOBER, 1852.

ART. I.—*Observations on Excision of the Inferior Maxillary Bone; Illustrated by Five Cases.* By S. D. GROSS, M. D., Professor of Surgery in the Medical Department of the University of Louisville.

In the last number of this Journal, I published a paper on excision of the upper jaw, preceded by a brief account of the more frequent and important diseases requiring this operation, and followed by a history of seven cases in which this bone was removed, either wholly, or in part. It is my intention, in the present communication, to offer some remarks upon exsection of the lower jaw, accompanied by several illustrative cases.

The maladies of the two jaws are so much alike, in their essential features, that the remarks which were made, in the preceding number of this Journal, respecting those of the one, may be received as strictly applicable to those of the other. Indeed, if there is any difference in this particular, it must be ascribed wholly to the modifying influence of the antrum of Highmore ; though it would be difficult to determine, in the present state of pathology, in what this influence consists, supposing it to be real and operative. All that we know, with any positive certainty, is, that medullary disease is much more frequent in the upper than the lower jaw, and the only means which we have of accounting for this circumstance, is the existence of the maxillary sinus, which, being lined by a mucous membrane, and charged with the execution of an important office, is peculiarly prone to take on this kind of action. The inferior jaw, on the contrary, is a solid structure, destitute of mucous tissue, and is, as all experience proves, particularly subject to fibro-cartilaginous, epuliform, and cystic formations.

My experience is that *encephaloid* of the inferior jaw is much more frequent in children and young adults than at any other period of life. Indeed, the very worst cases that I have seen of this disease occurred before the tenth year, and ran their course with a rapidity truly frightful. Most of the sufferers thus affected die within six or eight months from the commencement of the attack ; and if an attempt be made to relieve them by operation, the malady is almost sure to return in a very short time, either at the cicatrice, or in the adjacent structures, especially the lymphatic ganglions. The prognosis here, is, if posible, still more unfavorable than in medullary disease of the superior jaw.

The *epuliform* tumor is much more common in the lower jaw than in the upper; indeed, it appears to have a special predilection for this bone, springing up, generally, without any obvious cause, or under the influence of the most trifling irritation, and often acquiring a very extraordinary magnitude in a few weeks. In all the cases of this disease that have fallen under my observation, excision of the tumor, unless the operation embraced the bone at the site of the excrescence, was worse than useless. The malady has nearly always a most astonishing repullulating tendency, though the action which accompanies it is not always, perhaps, indeed, not generally, malignant in its character. Still, the proper rule of practice, in such a case, is to sacrifice a portion of the affected bone, taking care, as a means of safety, to cut it away some distance beyond the limits of the morbid growth. My fifth case affords a remarkable example of the reproductive, persecuting tendency, if I may so express myself, of this singular disease.

The *fibro-cartilaginous* tumor of the lower jaw is capable of acquiring an immense magnitude. Instances are recorded where it was as big as the patient's head, accompanied with the most hideous and disgusting distortion of the features. In general, it is slow in its growth, and unattended with contamination of the system and of the adjacent parts; circumstances in its history of the greatest diagnostic value: when removed it manifests no disposition to return. Examined with reference to its structure, it is found to contain, usually, a considerable number of cells, filled with various kinds of substances, serous, glairy, sanguineous, and purulent, surrounded and traversed by osseous spicula, and fibrous, fibro-cartilaginous, and cartilaginous septa. The compact structure is commonly absorbed, or softened and broken up; and, occasionally, the greater portion of the bone is converted into a hollow shell, separated into different compartments, and occupied by different fluids.

There is a peculiar tumor of the lower jaw, to which I have ventured, in my "Elements of Pathological Anatomy," to apply the term *hæmatoid*, as most expressive of its true character. It consists essentially in an expansion of the two lamellæ of the bone into a cavity, or hollow, occupied by dark coagulated blood, tolerably firm in its consistence, and evidently in a state of partial organization. The walls of the tumor are thin, but firm, sometimes elastic, at other times inelastic, and composed entirely of the compact tissue, invested by its periosteum. Its volume varies from that of a pigeon's egg to that of an orange. It is of slow formation, and is perfectly free from malignancy. A good specimen of this tumor is afforded in the case of Smith, the first on the list. I am not aware that this disease has ever been met with in the upper jaw.

The lower jaw is sometimes expanded, at one or more points, into a hard, firm, solid tumor, constituting a species of *hypertrophy*. The density of the affected part is occasionally equal to that of ivory. A few years ago, Dr. Pinkney, a highly intelligent surgeon of the United States Navy, showed me a piece of the body of the inferior maxillary bone, which he had removed for an affection of this kind from a man at Lima, and which was so hard that he found it almost impossible to divide it with the saw.

Excision of the lower jaw is sometimes required on account of *necrosis*. About five years ago I was consulted by a young man of the name of Bainbridge, from Cincinnati, respecting an affection of this kind, induced by the action of mercury, administered profusely, some months before, during a severe attack of remittent fever. He had suffered very much, and his mouth, gums, and jaw were in a horrible condition. Upon examination, I found a portion of the bone on the right side perfectly denuded, and as black as a coal; it was quite movable, and was easily extracted with a pair of bone-forceps, without any incision, except a slight internal one. It proved to be a part of the body of the jaw, with the ascending ramus, and the coronoid and condyloid processes. A case recently occurred in which the entire lower jaw was removed for an affection of this description, the surgeon making an incision from ear to ear in order to accomplish his object. It seems to me that in such a case the bone might be easily removed without any external incision whatever, simply by dividing it at the chin, and then extracting each piece separately with the forceps. Exsection, properly so termed, can hardly be required under such circumstances. If the parts be loose, as they generally are, or ought to be, before an attempt is made at exsection, a single cut will often suffice to liberate them. Where the necrosis is very extensive, or the exfoliation imperfect, a more free dissection will, of course, be necessary. The eclat of an operation should never induce a surgeon to inflict improper mutilation upon his patient. It is bad enough, in all conscience, for a man to lose his jaw, but to be, at the same time, unnecessarily disfigured, is unpardonable. The generous soldier never cuts off the ears, or pulls out the eyes of his enemy after he has vanquished him. He contents himself with the execution of an imperative and indispensable duty.

Portions of the inferior maxillary bone have been removed on account of *caries* of its substance, and even for malformation merely of the coronoid process, impeding the evolution of the wisdom tooth. The cases requiring such an operation, for such a cause, must be very rare indeed.

In concluding these brief remarks upon the nature of the principal affections which require amputation of the lower jaw, I wish to say a few words respecting the changes which the *coronoid and condyloid processes* are so apt to undergo in some of the abnormal growths of this bone. It is a singular fact, and one which does not appear to be generally known, that these processes are seldom the seat of any new deposit, whether benign or malignant. They seem to possess a remarkable conservative power. Nevertheless, there are few cases in which the bone is extensively involved, where they do not experience important alterations, not so much from any extension of the disease, as from the effects, apparently, of ordinary inflammation, developed under the influence of the irritation caused by the encroaching tumor. The most common changes which they undergo, so far as my observation enables me to judge, consist in their conversion into thick, irregular knobs, more or less porous and unhealthy; sometimes preternaturally firm, at other times quite soft and disorganized. The periosteum of the coronoid process and neck of the bone is generally, in such cases, considerably thickened, and the ligamentous connection of the temporo-maxillary articulation so much altered as to be scarcely distinguishable; the inter-articular cartilage is absorbed, or so adherent to the contiguous surfaces as to cause partial, if not complete anchylosis. Occasionally the coronoid and condyloid processes are very much wasted, or even entirely annihilated. Owing to these various changes, it is usually easy to effect the liberation of these processes, while the reverse is the case when they retain their normal character.

It is not necessary, at this late day, seriously to discuss the *claims* of any one to the credit of priority in this operation. This credit, and it is a credit of which any one may justly be proud, is now universally conceded, by all enlightened surgeons, both at home and abroad, to a Western physician, Dr. W. H. Deaderick, formerly of Rogersville, now of Athens, Tennessee. How this gentleman, who must have been very young at the time, came to undertake an operation which was not only formidable in itself, but without a precedent in the annals of surgery, is entirely a matter of conjecture. It may be supposed, however, that he was an excellent anatomist, and a bold, adventurous operator, who did not hesitate to think and to act for himself. Dr. Deaderick is still living. and long may he continue to enjoy the fruits of an honorable and well-spent professional life.

The case of Dr. Deaderick, requiring the operation under consideration, is too interesting and important to be passed in silence. The patient was a lad, fourteen years of age, who had a tumor of a cartilaginous structure, on the left side of the jaw, filling nearly the whole of the mouth, and causing great difficulty in swallowing, and even, occasionally, in breathing. The operation was performed on the 6th of February, 1810.* An incision was commenced under the zygomatic process, and carried across the tumor, in the direction of the lower jaw, to nearly an inch beyond the middle of the chin. From the centre of this, and, consequently, at a right angle with it, another incision was extended a short distance down the neck. The flaps thus marked off being separated from the diseased mass, the bone was sawed off immediately at the angle and centre of the chin. The wound was united in the usual manner, and the boy had a speedy and happy recovery; continuing perfectly well thirteen years after the operation.

The next operation of this kind upon the lower jaw was performed by Dupuytren, on the 30th November, 1812, without, it may be presumed, any knowledge, on his part, that he he had been anticipated in this undertaking by any one in America; for it is a singular fact that no account of Dr. Deaderick's case appeared in print until thirteen years after its occurrence, so regardless was this gentleman of any reputation that might be supposed to arise from its publication. In November, 1821, Dr. Mott, of New York, excised nearly the whole of the inferior jaw on one side; and, eighteen months afterwards, he removed all that portion of the bone which intervenes between the right temporo-maxillary joint and the second bicuspid tooth on the left side. This, so far as I can ascertain, is the first instance in which exarticulation was

* American Medical Recorder, vol. 6, p. 516.

attempted in the United States. Two years prior to this, Dr. Graefe, of Berlin, performed a similar operation ; and in 1816, Mr. Anthony White, of London, removed half a necrosed jaw from its socket.

From all that precedes, it is evident that Dr. Deaderick is entitled to the credit of having been the first surgeon that ever excised a *portion* of the lower jaw ; and, secondly, that Dr. Mott was the first, in this country, who romoved the bone at the temporo-maxillary articulation.

In my article on excision of the upper jaw, in the last number of this Journal, I had occasion to condemn, in a very pointed manner, the *ligation* of the primitive carotid artery, as a means of avoiding hemorrhage, in amputation of the maxillary bones. I recur to the subject here, because I find it stated, as late as 1846, in a work which is extensively read by the profession in the United States, that Dr. Mott lays it down as a principle, in all severe cases requiring extensive exsection of the lower jaw, to take up the primitive carotid artery as a preliminary and indispensable step, in order to cut off the dangerous hemorrhage which would otherwise ensue from the division of its main branches.* I do not wish to criticise the practice of Dr. Mott in a department of operative surgery, with the advancement of which his own name and fame are so intimately and so honorably associated ; but it does seem to me that it would have been well had he expressed himself with more caution on so important a subject. Whatever Dr. Mott may utter is received by certain physicians, especially by his own pupils, as oracular, and it is for this reason that I am induced to notice, and I do it with all possible candor and good feeling, the practice here referred to. I humbly submit whether it would not have been better if this eminent surgeon had confined him-

* Velpeau's Operative Surgery, by Townsend & Mott, vol. 2, p. 909: New York, 1846.

self to saying that *he* had found it necessary, in his more severe and formidable cases, to tie the carotid artery as a preliminary measure, than to assert that it was an "indispensable step," and that, therefore, it should always be adopted, under such circumstances, as a *principle?* The ligation of this vessel is, in general, easy enough ; but the consequences are what we are to look to, and these are not to be regarded too lightly. In one of Dr. Mott's cases, where the carotid artery was secured immediately before the disarticulation of one-half of the jaw, the hemorrhage was extremely profuse, and not less than from fifteen to twenty vessels required to be tied during and after the operation. In another instance, notwithstanding the greatest precaution was employed, the patient became so faint and exhausted, and the mind so much perturbed, during this "preliminary and indispensable" operation, that the excision of the tumor was obliged to be deferred until the following day. Dr. Warren, of Boston, in a similar undertaking, wounded a vein in searching for the carotid, and came very near losing his patient from the entrance of air into the circulation. The jaw was removed a week afterwards without ligating the artery. Mr. Lizars, of Edinburgh, in attempting to excise the superior maxillary bone for a medullary tumor of the antrum, commenced by tying the carotid on the affected side, but was prevented from proceeding by excessive hemorrhage, the patient having lost upwards of two pounds of blood in a few seconds.*

But the above are not the only objections to the performance of this operation in cases of excision of the jaw. Ligation of the primitive carotid artery is often followed by serious lesion of the brain, and by severe and even fatal inflammation of the air-passages. The inter-

*See Dr. Norris's interesting Statistics, Amer. Journal Med. Sciences, vol. 14, p. 31.

estingfacts and tables of Miller and Norris upon this subject are conlusive; and they should serve to admonish us never to resort to this operation, except under circumstances of the most positive and pressing character. To complicate, without any necessity, a case that is in itself already sufficiently severe and hazardous, is to augment the chances of a fatal termination, and to sport wantonly with human life. It ought to be sufficient glory for an operator, if glory he must have, to limit himself to the performance of what is strictly needful. To go beyond this, in any particular case, is to bring discredit both upon himself and upon the science of surgery.

In the only three instances in which I have removed the jaw at the temporo-maxillary articulation, I did not find it necessary, in two, to use a single ligature, and in the other, involving an immense tumor, not more than two or three small vessels were tied. These examples, so far as they go, confirm the opinion of Liston, Syme, and others, upon the propriety of omitting the ligation of the primitive corotid, as a preliminary measure in exsection of this bone.

It would be a waste of time to attempt here to lay down rules as to the best mode of *performing excision* of the lower jaw. The works on operative surgery now before the profession, are so numerous and so able as to preclude the necessity of any labor of this kind. There are a few points, however, upon which I deem it proper to comment, inasmuch as they are not generally dwelt upon by writers with sufficient emphasis; if, indeed, some of them have not been wholly overlooked.

One of the most important circumstances to be observed in exsection of the lower jaw, according to my experience, is to keep in close contact with the morbid structure, and yet sufficiently away from it to prevent any portion of it from being left behind. This would seem almost like a contradiction, and yet it is, in reality, anything but a contradiction. By attention to this rule, which I regard as of paramount importance, two great ends are attained: the easy removal of the tumor by a neat and rapid dissection, and the avoidance of hemorrhage. Cutting into the tumor is almost certain to be followed by the division of large vessels, which do not fail to bleed profusely, unless checked by compression or other means, until the operation is completed. Besides, chipping off a piece here and a piece there leads to a more or less tedious after-dissection, which is always attended with pain to the patient, and annoyance to the operator.

Another important rule is, to work, as much as possible, with the handle instead of the edge and point of the knife, especially when we are detaching the bone from the soft parts. Whenever it can be done, a portion of the periosteum should be saved, and there are few cases in which this membrane is so thoroughly involved in the disease as to render this impracticable. The part thus rescued is of great importance afterwards in filling up the void produced by the removal of the bone, at the same time that it prevents undue injury to the other soft structures.

The *external incisions* should always be made in such a manner as to avoid the unsightly appearance resulting from a large and exposed scar. For this purpose, when it is designed, for example, to remove the half of the jaw at the articulation, the knife should, as a general rule, be carried along the base of the bone, from the zygomatic process, about three-quarters of an inch in front of the ear, to the chin, and thence some distance up the midian line, or even as high up as the red margin of the lip. When the tumor is of immense size, two incisions are sometimes required, so as to include an elliptical portion of the soft parts; but, unless this is the case, or the skin is seriously involved in the disease, not a particle of integument should be sacrificed; for, during the healing process there is usually inordinate contraction, and hence if this circumstance is not attended to, great and disagreeable deformity will be the inevitable consequence. By making the perpendicular incision some distance in front of the ear, there will be little danger of wounding the temporal or external carotid artery, and the trunk of the portio dura. Sometimes, as when the disarticulation is effected with difficulty, a short horizontal incision, just below the zygomatic process, will greatly expedite our efforts; but, in general, this is unnecessary. The duct of Steno should always, if possible, be avoided, as it may usually be by being careful not to carry the knife too high up, or too far forward.

When the alveolar process alone is involved, the affected part may often be removed without any external incision at all, simply by detaching the lip or cheek from the bone, and holding it out of the way, while the osseous structures are divided with the saw. Conducted in this manner, the operation is productive of hardly any disfigurement.

When the operation involves the removal of the jaw at the joint, the best plan is to expose the tumor as rapidly and carefully as possible, and then saw the bone at the anterior limits of the morbid mass. This greatly expedites not only the process of disarticulation, but the separation of the jaw from its muscular and mucous attachments, as it enables the operator, by taking hold of its anterior extremity, to move the bone in any direction he pleases.

When the alveolar process alone is affected, or along with only a portion of the body of the jaw, it is a good rule, as was stated, long ago, by Dr. John Rhea Barton, of Philadelphia, to save the base of the bone, nay, even the merest strip of it, in order that it may serve as a support to the soft tissues, and become the nucleus of a new deposit.

One of the difficulties connected with this operation relates to the liberation of the coronoid and condyloid processes. The instrument which has always been employed for this purpose is the knife, or the knife and saw. The fibres of the temporal muscle, embracing the coronoid process on every side, are directed to be cut close to their attachments, or, instead of this, the process is sawn through at its base; the structures of the temporo-maxillary articulation are always divided with the point of the knife, entered at the anterior or posterior part of the joint, as may be most convenient. Now, it has always appeared to me that this mode of procedure should, if possible, be avoided, as it is apt to be followed by serious hemorrhage, and by injury of important nerves. This is especially the case with regard to the separation of the condyle, lying as it does in close and intimate relation with the internal maxillary artery, which must necessarily be endangered by the knife in this stage of the operation. A wound of this vessel, just as the operation is about to be finished, is an embarrassing circumstance, from the difficulty of applying a ligature, and is liable to be accompanied with copious hemorrhage. The coronoid process, although it projects up some distance into the zygomatic fossa, is separated with less difficulty, and, as it lies anterior to the maxillary artery, there is but little danger of interfering with this vessel. Still, a pretty smart hemorrhage occasionally results from the division, simply, of the little arteries of the temporal muscle.

To obviate this danger, as well as to expedite the process of disarticulation, usually, and, indeed, very justly regarded, in the ordinary mode, as no very easy part of the operation, I have used, with great advantage, an instrument combining the principles of a lever and a knife. The accompanying sketch will convey a much better idea of it than the most elaborate description. The blade is slightly curved upon the flat, and is three inches and a quarter in length, by three-eighths of an inch in width; its thickness is about one line and a third. Its free extremity terminates in a convex edge, beveled off in front and behind, so as to admit of being used for dividing the periosteum, or scraping the bone, as may be deemed necessary. The other extremity is set in a stout rough handle, nearly four inches long. A perfectly straight instrument of this kind may be used with much advantage.

In the only three instances in which I have had occasion to remove the jaw at the temporo-maxillary articulation, I have found, in two, both the condyloid and coronoid processes a good deal enlarged, apparently from simple hypertrophy, and the periosteum very considerably thickened; thereby allowing itself to be easily pealed off from the bone. Whenever this is the case, and my belief is that the occurrence is very frequent, the liberation of the parts in question may be readily effected with the instrument which is here delineated. All that is necessary is to insinuate the convex, or semi-blunt edge beneath the fibrous covering of the coronoid process, and after separating it for some distance, to prize the bone out of its place. In the same manner the soft structure may be detached from the condyle of the jaw, and the latter raised out of the glenoid cavity. The whole is the work of a few seconds, and the great beauty of the proceeding is, as before stated, its entire freedom from danger to the maxillary and other arteries, as well as the trunk and deep-seated branches of the portio dura. Where these processes with their investing structures are perfectly sound, the separation must be effected, at least in part, with the knife, but even here the instrument above described may afford valuable aid.

I have, within the last few days, had an opportunity of testing this instrument in a case of disarticulation of the jaw, unattended with the slightest change of structure in the condyloid and coronoid processes. The operation was unusually difficult, on account of the shortness of the affected bone, portions of which had been previously removed on two different occasions. After having dissected off the skin, the bone was thoroughly denuded in every direction, both of periosteum and muscles, with the instrument in question. The parotid gland was but little injured, and the duct of Steno and the trunk of the portio dura were, there was reason to believe, entirely intact. No important artery was wounded; a circumstance which could hardly have been avoided had the separation of the coronoid and condyloid processes been effected with the knife. The bone, at the close of the operation, was completely devoid of periosteum, and even of the cartilaginous incrustation of the condyloid process.

The *gap* left by this operation is occasionally, perhaps frequently, filled up, especially in young subjects, by a cartilaginous formation, of an irregularly cylindrical shape, which, while it serves to support the jaw in mastication, assists materially in reestablishing the symmetry of the features. The time required for the production of this substitute varies, it may be supposed, in different cases, from a few months to several years. Even when one-half of the bone has been removed, nature sometimes succeeds most admirably in her efforts. In 1832, I had an opportunity of seeing an Irish lad, aged about seventeen, from whom the late distinguished Dr. Cusack, of Dublin, had removed, four years previously, the left half of the inferior maxilla, on account of a fibro-cartilaginous affection. In this instance, nature had made an attempt at reproduction, the part being replaced by a thick, rounded piece of cartilage, sufficiently strong to subserve the ordinary purposes of mastication. It is astonishing what little deformity of the features there was in this case.

CASES OF EXCISON OF THE LOWER JAW.

*Case* 1.—David L. Smith, thirty-five years of age, a farmer, of thin, spare habit, from Ohio county, Kentucky, visited Louisville early in October, 1844, on account of an affection of his lower jaw. The disease was first noticed, about three years ago, in the form of a hard, solid tubercle, not larger than a hazel-nut, on the left side, just behind the cuspid tooth. Its progress was very slow until last Christmas, when it began to increase with considerable rapidity; and it soon became the seat of a constant pain, of a dull, aching character. The malady made its appearance without any assignable cause. The patient had never sustained any injury of the part affected; and his general health had always been good, with the exception of an occasional attack of dyspepsia.

When Smith placed himself under my care, the tumor was exceedingly hard and firm, and bulged forward in such a manner as to cause considerable deformity of the left side of the face. It extended from the lateral incisor on the right to the middle of the large grinder on the left. The corresponding teeth inclined backwards and inwards, and were so loose as to be unfit for the purposes of mastication. The gum was of an unnaturally red color, and somewhat hypertrophied, but otherwise perfectly healthy. The mucous membrane and cheek were perfectly sound, and there was no enlargement of any of the surrounding lymphatic ganglions.

Excision of the tumor was performed on the 10th of October, in the presence of Professor Miller and Drs. Johnston, Cochran, T. L. Caldwell, Colescott, Bayless, and Somerby, the distinguished dentist. The patient sat upon a chair with his head supported upon the breast of an assistant; and, as a preliminary step, Dr. Somerby extracted the first large grinder on the left side and the right lateral incisor. A rectangular incision was then made, commencing at the free margin of the lower lip, a little to the right of the median line, and extending down to the base of the chin, and from thence backwards, along the outer and inferior surface of the jaw, as far as the posterior limits of the disease. The flap thus formed was carefully dissected off from the tumor, and held out of the way, while the bone was sawed through, first, on a level with the right canine tooth, and secondly just in front of the middle grinder on the left side. The coronary artery was controlled by the fingers of an assistant. In consequence of the excessive hardness of the bone, great difficulty was experienced in dividing it; this, however, was at length accomplished with a saw constructed for the purpose, and the tumor removed. In pulling the tumor downwards, a portion of its posterior surface, a mere shell, broke off, and, as it was perfectly sound, was left behind.

The edges of the wound were brought together by four twisted sutures, the first of which was introduced near the red margin of the lip. No vessels required to be tied, and scarcely three ounces of blood were lost. A piece of lint was placed within the mouth to support the chin, and opposite to this, on the outside, a compress; the whole being confined by a roller carried round the head and jaw.

For several days after the operation, Smith had some difficulty in swallowing, and a considerable swelling of the lip, with a copious discharge of saliva, and some febrile commotion. These symptoms speedily yielded to an active cathartic and cooling drinks. At the end of the fourth day, the three lower needles were removed, and on the fifth the upper; the union was complete. Suppuration ensued in forty-eight hours in the track of the bone, granulations soon began to form, and in less than three weeks the mouth was perfectly healed. The cicatrice is scarcely perceptible; and, with the exception of a slight retrocession of the chin, there is no deformity.

The tumor, about the volume of a medium-sized orange, was found to consist of a mere osseous shell, without any vestige of the cancellated structure, and was occupied by three red, solid coagula, the largest of which did not exceed the volume of a pigeon's egg. The cavity was only partially filled by the clotted blood, which adhered to the inner surface of the bony wall, and was evidently organized. I have repeatedly heard from Smith since the operation, and am happy to say that there has been no return of his disease, which was certainly not malignant.

**Case* 2.—Kitty, a negress, nine years of age, was brought to me for professional advice, on the 2d of May, 1846, by her master, Mr. Lawton, of Rumsey, Kentucky. Eighteen months previously she punctured the left side of her face with a piece of wood, which she happened to have in her mouth as she fell, head-foremost, from a high fence. Some inflammation ensued, but this rapidly disappeared, and nothing further was thought of the accident until four months after, when a slight enlargement was noticed at the original site of the injury. This gradually extended until it involved the whole of the left side of the jaw, from the ear to within a few lines of the symphysis of the chin, and from the middle of the cheek to the upper part of the neck. The tumor, which had increased very much within the last three or four weeks, was very prominent externally, and reached, at the time of my examination, when the head was held in its ordinary position, nearly down to the clavicle. Articulation and mastication were both very much impeded, and the jaw was so completely anchylosed that it was almost impossible to move it in any direction; indeed, the interval

* American Journal Medical Sciences, vol. 16, p. 344.

between the dental arches was so small as not to permit the protrusion of the tip of tongue. The swelling was very hard externally, and towards the base of the jaw were two small points, which seemed to have been the seats of little abscesses, probably of a scrofulous character. In other respects the skin was perfectly sound. Internally, the bone appeared to be healthy, and did not encroach upon the tongue. The tumor was at first free from suffering, but during the last month, the girl complained of a good deal of pain, especially at night. The general health was unimpaired.

Such being the condition of the parts, it was obvious that nothing short of excision of a portion of the jaw could afford any relief. The operation was performed on the 5th of May, in the presence of Dr. Colescott and Dr. Thomas L. Caldwell, and a number of medical students. As a preliminary step, the body and limbs of the little patient were surrounded by a strong apron, carefully pinned behind, as in the operation for harelip. She was seated upon a chair, with her head inclined a little backwards, and supported by an assistant. An incision was made through the middle of the lower lip to the base of the chin, and, at a right angle with this, another along the body of the diseased bone. The flap thus marked out was then dissected up, and the jaw sawed through a little obliquely, immediately by the side of the symphysis, so as to preserve the two incisors. An incision was then carried from the tragus of the ear to the termination of the horizontal one, already mentioned; the integuments were rapidly dissected up; the masseter, temporal, mylohyoid, and pterygoid muscles were successively separated from their attachments; and the bone was finally raised from its socket. Not a little difficulty was experienced in this stage of the operation, from the cartilaginous adhesions between the coronoid and zygomatic processes, the former of which was very much thickened, and forced the latter out in such a manner as to render the cheek quite prominent, in that situation. The temporo-maxillary joint was completely anchylosed, and the attachments formed by the condyle rendered the disarticulation almost impracticable.

A part of the sub-maxillary gland appeared to be changed in structure, and was accordingly removed. Several lymphatic ganglions of the neck were, for the same reason, excised. The operation was exceedingly painful ; and, as I was about to disarticulate the bone, the patient swooned away. Scarcely six ounces of blood were lost, and, what is remarkable, not a single vessel required to be tied. The bleeding from the facial artery, which was divided in the early stage of the operation, was readily controlled by the finger of an assistant.

The tongue, stylo-hyoid muscle, and submaxillary gland were fully exposed by the dissection ; the parotid lay at the posterior part of the wound, and the external carotid was seen pulsating just below the ear.

The operation being over, the wound was kept covered for nearly three hours with a thick linen compress, frequently wet with cold water. At the expiration of this period, the edges were brought together by six twisted sutures, and a few adhesive straps, the whole being supported by a thick compress and a roller.

Very little pain was experienced after the operation, and the patient slept comfortably during the succeeding night. On the following day there was slight fever, with some swelling on the left side of the face, but this yielded speedily to a dose of Epsom salts and cooling drinks. The needles were removed at the end of the third day, and the parts were found to be beautifully united, both externally and internally. Subsequently the temple and cheek were invaded by erysipelas, brought on, apparently, by premature exposure and improper eating. A dose of calomel, and the application of tincture of iodine, promptly put a stop to this disease ; and from this time on, the convalescence was rapid and uninterrupted.

The tumor, on dissection, was found to be of a fibrous character, dense, inelastic, and of a dull whitish color. The periosteum, from which it probably arose, was considerably thickened, and could be peeled off, without difficulty, from the subjacent bone. The bone itself was considerably enlarged, and a section of it exhibited a uniform, compact surface, with here and there a small elongated cell. The coronoid and condyloid processes were remarkably thick, and the latter had lost its smooth and polished appearance. The body of the bone contained a perfectly healthy molar tooth.

Upwards of six years have now elapsed since the above operation, and there is, thus far, no evidence whatever of any tendency to a relapse of the disease for which it was undertaken. Kitty's master has recently informed me that she is entirely well, that there is hardly any deformity of the face, and that she chews, articulates, and swallows with as much facility as any one.

*Case* 3.—Milton, a colored man, aged 38, the father of four children, and a servant of Henry C. Ewin, Esq., of Todd county, Kentucky, was sent to me in April, 1850, on account of an enormous tumor of the left side of the lower jaw, which had latterly become a source of great suffering and annoyance to him, so much so, indeed, as to disqualify him for active exertion, and deprive him, almost wholly, of the power of mastication. The morbid growth had existed about twelve years, during the last four of which it had increased very rapidly, giving rise to the most hideous and disgusting deformity, and rendering the poor fellow an object of commiseration with every one who beheld him.

The dimensions and limits of the tumor, upon the arrival of the patient in Louisville, were as follows: From the angle of the mouth, in a straight line to the ear, nine inches; from the chin to its posterior extremity, eleven inches and three-quarters; and in the vertical diameter, ten inches and a half. Terminating an inch and a half behind the left ear, it extended above, on a level with the zygomatic process and ball of the eye, and below to within about two inches of the clavicle, when the head was forcibly bent back, while it almost touched that bone when the head was in its natural position. It overlapped the upper part of the thyroid cartilage, and dragged the mouth so much over to the right side, that the left angle of this outlet was habitually on a line with the septum of the nose. The outer surface of the tumor was quite uniform, except towards its base, where two small openings existed, giving vent to a thin, viscid, slightly purulent fluid, and environed each by a well-marked cicatrix ; here and there was a softish and elastic spot, and just in front of the ear it imparted a sensation as if it contained a fluid.

Viewed internally, the morbid growth was found to extend beyond the median line, throwing the tongue, which reposed, as it were, upon its upper surface, completely upwards against the roof of the mouth, and rendering it almost impossible to protrude it beyond the lips. Posteriorly the tumor reached tothe arch of the palate, greatly diminishing the opening of the fauces, and seriously embarrassing deglutition, articulation, and even respiration. Its inner surface was smooth, shining, and elastic, and was marked by a groove, caused by the grinders of the superior jaw.

When the tumor was first noticed, and for a long time afterwards, it was free from pain and even uneasiness; during the last two years, however, it was the seat of a constant aching feeling, and not unfrequently of a keen, darting pain. The general health had been good ; but latterly the man had lost strength and flesh, and was unable to sleep well at night. No cause could be assigned for the origin of the tumor.

Such was the frightful condition of my patient after he had been for some time under my care, that I was greatly in doubt whether an attempt should be made to rid him of his burden. An eminent professional gentleman who saw him with me, about ten days after his arrival in Louisville, in fact, strenuously opposed the operation, on the ground that it would almost certainly be followed with fatal results, if not at the time, at all events very soon after, and that it would thus tend to bring surgery into discredit. The impression which the interview made was not without some weight; but my feelings for the poor fellow at length overcame my scruples, and I determined to afford him a chance for his life. He had undergone ample preparatory treatment, and was therefore in excellent condition for the knife.

The operation was performed on the 13th of May, at the amphitheatre of the Louisville Marine Hospital, in the presence of my friend Dr. Lopez, of Mobile, Alabama, and a number of physians and students. Professor Miller, Dr. Goddard, the dentist, Dr. T. G. Richardson, and my pupils, Dr. Thomson and Dr. Trabue, kindly afforded me their assistance. The patient, placed under the influence of chloroform, lay upon the table, with his head resting upon a pillow, and his face inclining towards the right side. The left central incisor having been extracted by Dr. Goddard, I made an incision over the prominent portion of the tumor, commencing half an inch in front of the ear, and just beneath the zygomatic process, and extending forwards in a semi-circular direction as far as the centre of the chin, and thence upwards along the middle line through the free margin of the lip. Another incision, beginning near the posterior extremity of the first, was then carried forwards for some distance over the central portion of the swelling, so as to include the unsound skin in the form of an ellipsis. Several vessels, among others the external maxillary artery, were divided, and immediately compressed by the fingers of my assistants. Detaching the lip and integuments of the chin, I at once divided the jaw at the symphysis with a metacarpal saw; a procedure which occupied hardly twenty seconds, and which greatly expedited the future steps of the operation. Turning my attention now to the flaps, I turned one up and the other down, by bold and rapid strokes of the scalpel, and thus exposed the main mass of the diseased structure. By the time this was accomplished, the patient was pulseless, and it was necessary to wait five or six minutes, and give him brandy and ammonia before he was sufficiently restored to enable me to proceed. I next dissected the tumor from its attachments to the mucous membrane of the mouth, cut the mylo-hyoid, genio-hyoid, genio-hyoglossal, masseter, and pterygoid muscles, and separated the coronoid and condyloid processes, by means of a curved lever, with a strong handle, and a rounded, somewhat sharp extremity. This stage of the operation was executed with great facility; the ligaments and temporal muscle offering hardly any resistance.

Scarcely half a pint of blood was lost. The only arteries requiring the ligature were the external maxillary and the inferior dental, together with a small muscular branch. The external carotid and the internal maxillary were seen pulsating at the lower and back part of the wound, which was of great extent. The temporal artery was not divided.

The patient being still faint at the close of the operation, it was necessary to retain him on the table, and to administer stimulants, as brandy and ammonia, with sinapisms to the extremities. As soon as he was sufficiently recovered, I brought the parts together with six twisted and two interrupted sutures, filled the vacuities in the cheek with patent lint, and placed a compress upon the side of the face, in the line of the late tumor; the whole being supported by a roller carried round the head and chin. The patient was then put in bed and ordered one grain of sulphate of morphia.

In the afternoon, at seven o'clock, the patient was quite comfortable; he had no pain or fever, and swallowed with perfect ease. The morning after the operation I found him in fine spirits, and I learned that he had slept well during the previous night; he complained of slight stiffness in the throat, and had a little swelling in the face, but hardly any fever or thirst. He was ordered a dose of oil, which produced several slight evacuations during the preceding night, which he passed very comfortably.

The dressings were taken off on the 16th, when the wound was found united in its entire extent, except for about an inch at its centre, where it gaped a little. The parts here were brought together by two sutures, the four front needles taken out, aud nearly all the lint removed from the mouth. There was hardly any swelling or suppuration, and the patient swallowed well, and had a good appetite. The other sutures and lint were removed on the 19th; when the patient moved his tongue well, and performed deglutition with great facility. In a word, he was perfectly comfortable, and entirely out of danger.

On the 13th of June, Milton left Louisville on the steamer Mayflower, for Nashville. He was still feeble, but could walk several miles a day. The left side of the face was somewhat swollen and tender, especially towards the zygoma; and a small opening still existed about the centre of the cicatrice, which was considerably depressed, or sunk in towards the cheek. Through this opening a little saliva flowed during eating. A minute fistula, as big as a pin's head, also existed over the parotid region, and occasionly discharged a drop of saliva. The patient had an excellent appetite, and had some use of the right side of the jaw in mastication.

From a letter received from Mr. Ewin, dated July 30, 1852, I learn that Milton is in good health, and that his face is entirely healed and sound. "He is regaining his flesh, and looks well, but is not able to bear hard labor, or exposure to the hot sun."

The tumor was carefully examined after removal, and was found to consist chiefly of a number of osseous cysts, of variable dimensions, and filled with a thin, turbid serum. None of the cells were occupied with encephaloid or scirrhous matter. When struck with the handle of a knife, the tumor emitted a sound like that of a dice-box. The bone was entirely sound in front, and also at the condyle and coronoid process.

*Case* 4.—A very genteel mechanic, thirty-five years of age, from the interior of the State of New York, consulted the late Professor Pattison and myself in November, 1850, for a fungous growth of the lower jaw, which he had first noticed not quite a year ago, and which had been removed some months before by a physician in the country. The tumor, which was of a pale reddish complexion, and of a dense, gristly consistence, sprung from the alveolar process, in the situation of the right molar teeth, the anterior of which was imbedded in its substance. It was about the size of an almond, and was the seat of a dull, heavy, aching pain, attended with some stiffness of the temporo-maxillary articulation, and more or less uneasiness in mastication. The integuments over the tumor were sound, and there was no perceptible external deformity. The general health was excellent.

The operation was performed on the 2d of November, at the Surgical Clinique of the University of New York, in presence of the medical class, and with the aid of Professor Pattison, the patient being under the influence of chloroform. The first bicuspid and last molar teeth having been removed, a horizontal incision, about three inches and a half in length, was made along the inferior margin of the body of the jaw, directly over the diseased part. The flaps were then dissected off, and the bone, which was excessively hard and dense, sawed through at the ramus posteriorly and just behind the mental foramen anteriorly. The facial artery was necessarily divided, and this, with a small muscular branch, was the only vessel that was tied. The edges of the wound were then brought in apposition, and retained by four twisted sutures, a compress, and a roller. Hey's metacarpal saw was employed for dividing the bone.

The patient had no untoward symptoms after the operation, except a slight attack of erysipelas, and went home in about two weeks perfectly well. The fungous growth, previously alluded to, had its origin deep in the alveolar process, and seemed to be of the nature of epulis. The base of the body of the bone was quite sound.

*Case* 5.—Mr. S. W. S., of Allen county, Kentucky, aged 32, farmer, tall and slender, and of florid complexion, consulted me in April, 1851, respecting an affection of the lower jaw, which, as he informed me, he first observed three months ago. The first thing that attracted his attention was a soreness of the gum round the last grinder of the lower jaw on the right side, which was soon succeeded by a small, red, tender swelling, most conspicuous on the inside of the tooth. The tumor gradually increased in volume, and as it did so the soreness, previously complained of, entirely disappeared. As he has never sustained any local injury, he supposes that the disease had a spontaneous origin. He was always healthy until his seventeenth year, when he was seized with some obscure affection of the heart, accompanied with neuralgic pains in the left side, which continued for a few months, and then gradually left him. Since that period he has never been sick.

When the swelling was the size of the end of the thumb, which it attained in a fortnight after it was first observed, it was cut out by a physician in the country. The operation, slight as it was, was attended with a good deal of hemorrhage. In two weeks, the tumor, which was now fully three times as big as before, was removed a second time, and along with it the last grinder, the root of which was much diseased. There was again a considerable hemorrhage. This operation was followed, in a few hours, by swelling of the corresponding side of the neck and face, which, rapidly increasing, became excessively painful, and at length terminated in suppuration. The discharge of matter was copious, and lasted for several weeks. When the patient arrived in town, April 23, the parts were still hard and rough, and there was a small fistulous opening just below the angle of the jaw, giving vent to a thin gleety fluid. The tumor, at this time, formed two large cylindrical masses, one on each side of the dental arch, of a pale red color, devoid of pain, elastic, and of a firm consistence, extending from the ramus of the jaw to the first bicuspid tooth. The cheek was sound, and was not in the least protruded by the morbid growth, which, however, considerably impeded the movements of the jaw, mastication, and articulation.

Satisfied, from all the circumstances of the case, that nothing short of excision of a portion of the jaw would hold out any prospect of permanent relief, I performed the operation on the 27th of April, Drs. Goddard. T. G. Richardson, and Thomson assisting. The anterior bicuspid being extracted, and chloroform administered, an incision, about four inches in length, and slightly curved in its direction, was made along the base of the jaw from before backwards; the parts, which were very hard, and bled quite freely, were then dissected up, and the bone thoroughly exposed at the seat of the tumor. Not knowing how deeply the disease extended, and anxious to save as much of the bone as possible, I limited myself, in the first instance, entirely to the alveolar process, but after it was removed, a careful examination rendered it plain enough that a portion of the morbid growth had been left behind. I determined, therefore, to saw the jaw through at the first bicuspid tooth, and just in front of the ascending ramus. Four ligatures were applied; among them, one to the external maxillary artery, which was inclined to bleed quite freely, as it could not possibly retract, in consequence of the indurated and condensed condition of the soft parts. The wound was dressed with seven twisted sutures, a compress, and roller. Upon inspecting it at the end of the fourth day, it was found to have united perfectly by the first intention. A little erysipelas made its appearance about thirty-six hours after the operation, but soon yielded to a dose of purgative medicine and the application of the tincture of iodine. No further trouble occurred, and the patient went home in about a fortnight after the operation.

In the latter part of June Mr. S. wrote me that he was doing well, except that he had some difficulty in masticating, and that there was still some swelling under the jaw. Early in September I heard from him again, to the effect that the gap left by the removal of the bone, was occupied by a tolerably large mass, quite dense and discharging constantly more or less pus. The tumor was steadily increasing, but was free from pain and soreness. At my request, he came to town a short time after; and on the 24th of September I operated upon him a second time, removing the whole of the new growth, which was about the size of a pullet's egg, and about three-quarters of an inch of the anterior extremity of the ramus of the jaw, from which the diseased structure seemed to spring. The incisions which it was necessary to make for this purpose, extended in a vertical and horizontal direction, from the outer part of the lower lip on the right side to the angle of the jaw, and were approximated with the twisted suture. In consequence, however, of the want of support in the cheek, a small part of the horizontal portion of the wound refused to unite; and it became necessary, afterwards, to pare the edges of the opening and to stitch them together, to facilitate adhesion. Notwithstanding this, however, it was not until sometime in April, 1852, that the consolidation was completed. On the 2d of July he wrote me that he was entirely well, and that there was not the slightest appearance of return of the local affection.

There can be no doubt that the tumor in this case was an epulis, commencing in the gum, or the gum and socket of the last molar tooth, and gradually extending to the alveolar process and body of the bone. Its reproductive tendency would mark it as malignant, and as likely to lead, at some future period, to disastrous results.

On the 21st of August last, Mr. S. wrote me that the disease was returning; and before I had time to answer his letter, he arrived in town. About three weeks before the date here referred to, a tumor began to form at the anterior extremity of the ramus of the jaw, which has rapidly increased until it is now the size of a pullet's egg, pressing against the upper grinders, and gently pushing out the posterior part of the cheek; it is of a rounded shape, very dense and firm, of a pale color, and quite tender on pressure. At times, sharp, stinging pains shoot through it. The front of the jaw is sound, and the general health excellent.

On the 31st of August, assisted by Drs. T. G. Richardson and Thomson, and my pupil, Mr. White, I excised the ramus of the jaw at the articulation. An incision, slightly curved in its direction, nearly four inches in length, and situated about nine lines in front of the ear, being carried from the zygoma to a short distance below the anterior extremity of the tumor, the flaps were detached from the subjacent structures, and the bone denuded with the instrument described in a previous part of this paper, aided by the point and handle of the scalpel. In the effort of separating the coronoid and condyloid processes, a pretty copious hemorrhage ensued, which was increased, for a few seconds, by the pressure which I directed to be made upon the common carotid artery, under the supposition that it was arterial in its character, which, however, was found not to be the fact. As soon as the bone was detached the bleeding ceased, and not a single ligature was required. Nearly a pint of blood was lost; and at one moment a considerable quantity passed into the patient's mouth, causing some embarrassment of breathing. The edges of the incision were carefully approximated by five twisted sutures, and supported by a compress and bandage; a small roll of patent lint, wet with a strong solution of alum, having been previously inserted into the upper angle of the wound.

The bone, examined after its removal, was found to be completely divested of its periosteum, and even of the cartilaginous covering of its condyle. Not a fibre of the masseter, temporal, and internal pterygoid muscles was left. A portion of the insertion of the external pterygoid alone was visible. With this exception, the bone, in the healthy part of its extent, was as clean as if it had just come out of the macerating tub. The morbid growth, scarcely as large as a pullet's egg, and of a dense, firm structure, partly fibro-cartilaginous, and partly osseous, was attached to the inner surface and anterior extremity of the bone, the latter of which was unnaturally thin and somewhat notched. The external surface, neck, condyle, and coronoid process were all sound; but the inner surface, nearly as far back as the dental foramen, was slightly roughened and thickened, from the effects of disease. The length of the bone, measuring from the condyle, was two inches and five-eights.

Some swelling of the cheek and lip supervened; but the pain was very slight, and the patient had, at no time, any difficulty in swallowing. The sutures were removed at the end of the fourth day, when the wound was found to have united by the first intention. The lint, at the upper angle of the wound, was allowed to remain till the sixth day. Two weeks have now elapsed since the operation, and the patient is perfectly well, having never had a single bad symptom. His general health is excellent.

## Figures and Tables

**Figure f1:**